# A Japanese traditional medicine Hochuekkito promotes negative conversion of vancomycin-resistant Enterococci

**DOI:** 10.1038/s41598-021-90890-4

**Published:** 2021-05-28

**Authors:** Junko Kohno, Tsuyoshi Kawamura, Akiko Kikuchi, Tetsuya Akaishi, Shin Takayama, Tadashi Ishii

**Affiliations:** 1 Department of Obstetrics and Gynecology, Hachinohe City Hospital, 1-1-3 Tamukai, Hachinohe, 031-8555 Japan; 2 Department of Neurosurgery, Hachinohe City Hospital, 1-1-3 Tamukai, Hachinohe, 031-8555 Japan; 3 Department of Education and Support for Regional Medicine, Tohoku University Hospital, 1-1, Seiryomachi, Aoba-ku, Sendai, 980-8574 Japan; 4 Department of Kampo Medicine, Tohoku University Hospital, 1-1, Seiryomachi, Aoba-ku, Sendai, 980-8574 Japan; 5 Department of Kampo and Integrative Medicine, Tohoku University Graduate School of Medicine, 1-2, Seiryomachi, Aoba-ku, Sendai, 980-8575 Japan

**Keywords:** Diseases, Medical research

## Abstract

Vancomycin-resistant enterococci (VRE) are prominent causes of nosocomial infections. Japanese traditional (Kampo) medicine promotes intestinal immunity and protects against bacterial infections. We assessed potential differences in the clinical course of VRE-positive patients, based on their characteristics and treatment with Kampo medicines. This retrospective observational study collected data from VRE-positive patients from August 2018 to July 2019 at a tertiary-care hospital in Japan. The data of 122 consecutive VRE-positive inpatients were analyzed. Sixty-nine patients were treated with probiotics, among whom, 18 were further treated with Kampo medicines. Twenty-six of the 122 patients subsequently died. In univariate analyses, subsequent VRE negative conversion significantly reduced the mortality of VRE-detected patients (*p* = .0003). Administration of probiotics (*p* = .0065) and Kampo medicines with probiotics (*p* = .0002), especially of the Kampo medicine hochuekkito (*p* = .0014), and a higher serum albumin level positively contributed to the subsequent VRE negative conversion. Multivariate analyses demonstrated that Kampo medicines and body mass index contributed to VRE negative conversion. Hochuekkito shortened the time needed for VRE negative conversion (*p* = 0.0485). Administration of Kampo medicines, especially of hochuekkito, in addition to probiotics in VRE patients may promote VRE negative conversion.

## Introduction

Vancomycin resistance makes it difficult to control and treat infectious diseases, particularly in immunocompromised patients. Recently, vancomycin-resistant enterococci (VRE) have been prominent causes of nosocomial infections. The first clinical VRE isolation in Japan was reported in 1996^1^. Subsequently, a nosocomial VRE outbreak occurred in 2005 in Kyoto^2^. Since then, sporadic hospital-acquired VRE infections have been reported in Japan. Recommended strategies for nosocomial infection control of VRE are early detection by screening, spread monitoring in-hospital, thorough prevention of contact transmission, and relevant isolation^3^. Few methods have been established for eliminating VRE colonization, but a recently published retrospective study suggested that certain probiotics can reduce its burden. Borgmann et al. reported that treatment with *Saccharomyces boulardii* and *Escherichia coli* reduce the transmission of VRE among patients in an early rehabilitation ward^4^. *Lactobacillus rhamnosus* GG administered to children may eliminate the gastrointestinal VRE carrier state^5^. However, in adults with comorbidities, *Lactobacillus rhamnosus* GG was not effective for reducing VRE colonization^6^. Therefore, the effect of probiotics as VRE treatment still requires further research.

Conversely, traditional Japanese (Kampo) medicines, which originate from ancient China, are used for the treatment of various diseases and conditions^7–9^. Kampo medicines generally consist of several crude drugs and include various bioactive compounds including flavonoids, alkaloids, triterpenoid saponins, and lactones. Certain Kampo medicines have been shown to promote intestinal immunity and protect against bacterial infections^10–12^. Regarding methicillin-resistant *Staphylococcus aureus* (MRSA), Nishida reported that hochuekkito (HET), a Kampo medicine formulation composed of 10 types of crude drugs and used to treat disease and stimulate immune function, significantly reduced the urine bacterial count 10 weeks after administration^10^. Matsui et al.^12^ reported that HET could inhibit MRSA growth and extend the survival time of MRSA-infected mice. Nakae et al. reported that juzentaihoto (JTT) led to negative bacterial cultures in five cases of refractory multidrug-resistant bacterial infection, including a MRSA case with severe burns^13^. These studies indicated that HET and JTT could be effective for reducing MRSA colonization. Particularly, HET was found to support the immune system by inducing anti-inflammatory responses based on intestinal conditions. However, the effect of Kampo medicines on patients with VRE colonization has not been investigated. We herein report an outbreak of VRE at a tertiary-care hospital in Japan. We aimed to assess potential differences in the clinical course of VRE colonization based on patients’ characteristics and treatment with either probiotics or Kampo medicines.

## Results

### Patient background affected mortality

In a total of 122 patients, VRE were detected in culture tests from sputum (n = 1), bile juice (n = 1), wound exudate (n = 1), urine (n = 3), and stool (n = 116). Clinical data on admission before VRE detection showed that 28 of the 122 cases considered (23.0%) were perioperative cases. The use of antibiotics after admission and before VRE detection was recorded in 110 of the 122 cases (90.2%). As for the nutritional route, 54 of the 122 patients (44.3%) were fed through a nasogastric feeding tube (Table 1). During the subsequent clinical course of the 122 VRE-positive patients, 26 patients died and 96 survived. Sixty-nine patients were treated with probiotics, and 18 patients further received Kampo medicines in addition to probiotics (16, HET; 1, JTT; and 1, ninjin’yoeito (NYT)). Fifty-three patients received neither probiotics nor Kampo medicines. Four patients were diagnosed with infection due to VRE. Three of them died, while one recovered and was moved to another hospital. Only one deceased patient, who had suffered from severe burns, had undergone treatment with linezolid. *Enterococcus faecium* accounted for all VRE infections in this cohort. The genotype of all examined samples was *Van A*. No probiotic-related adverse events were observed in this study. One patient treated with Kampo medicines showed hypokalemia but soon improved with the administration of 25 mg of spironolactone.

**Table 1:** Comparative analysis of clinical and therapeutic parameters according to subsequent mortality during hospitalization.

	Deceased (n = 26)	Survived (n = 96)	*p*-value
Male:Female (n)	15:11	58:38	0.8015
Age*	69.9 ± 17.5 years	72.6 ± 12.2 years	0.3658
Body mass index at hospitalization*	22.94 ± 4.02	22.16 ± 4.47	0.4299
Serum albumin level at hospitalization*	2.75 ± 0.85 g/dL	3.23 ± 0.78 g/dL	0.0087
Length of hospitalization since VRE positivity^†^	22 (7–56) days	35 (15–78) days	0.1094
**Medical history**			
Diabetes mellitus, n (%)	7 (26.9%)	31 (32.3%)	0.8117
Hypertension, n (%)	9 (34.6%)	41 (42.7%)	0.5070
Dyslipidemia, n (%)	1 (3.8%)	12 (12.5%)	0.2955
Cardiovascular disease, n (%)	7 (26.9%)	16 (16.7%)	0.2625
Malignant tumor, n (%)	3 (11.5%)	28 (29.2%)	0.0787
**Primary causative disease of admission**			
Admission with cerebral stroke, n (%)	4 (15.4%)	21 (21.9%)	0.5898
Admission with traumatic injury, n (%)	4 (15.4%)	11 (11.5%)	0.7362
Admission with total infectious diseases, n (%)	13 (50.0%)	31 (32.3%)	0.0953
Admission with pneumoniae, n (%)	7 (26.9%)	12 (12.5%)	0.1224
**Details of treatment before VRE detection**			
Perioperative cases (n; surgery after admission [%])	5 (19.2%)	23 (24.0%)	0.7938
Use of antibiotics (total; n [%])	25 (96.2%)	85 (88.5%)	0.4579
Use of antibiotics (VCM; n [%])	11 (42.3%)	15 (15.6%)	0.0032
Use of a nasogastric feeding tube (n [%])	14 (53.8%)	40 (41.7%)	0.2674
**Details of the treatment for VRE**			
Probiotics, n (%)	18 (69.2%)	51 (53.1%)	0.1823
Total Kampo medicines, n (%)	1 (3.8%)	17 (17.7%)	0.1170
Hochuekkito, n (%)	1 (3.8%)	15 (15.6%)	0.1885
**Subsequent achievement of VRE negative conversion**			
Achieved, n (%)	1 (3.8%)	38 (39.6%)	0.0003

Kampo medicines (total) means hochuekkito (n = 16), juzentaihoto (n = 1), and ninjin'yoeito (n = 1).

*VCM* vancomycin, *VRE* vancomycin-resistant enterococcus.

*Mean and standard deviation, †median and interquartile range.

First, we examined the factors that affected the mortality of VRE-positive patients by univariate analyses (Table 1). The demographic, clinical, and therapeutic data were analyzed. None of the evaluated demographic, pre-onset clinical or therapeutic factors significantly affected mortality, except for the serum albumin level at hospitalization. On the other hand, the subsequent VRE negative conversion significantly reduced the mortality of patients with VRE colonization (*p* = 0.0003). Prior vancomycin (VCM) use before confirmation of VRE positivity in the stool negatively affected mortality (*p* = 0.0032). These data are summarized in Table 1.

### Impact of considered factors on the overall survival rate

**Figure 1:**
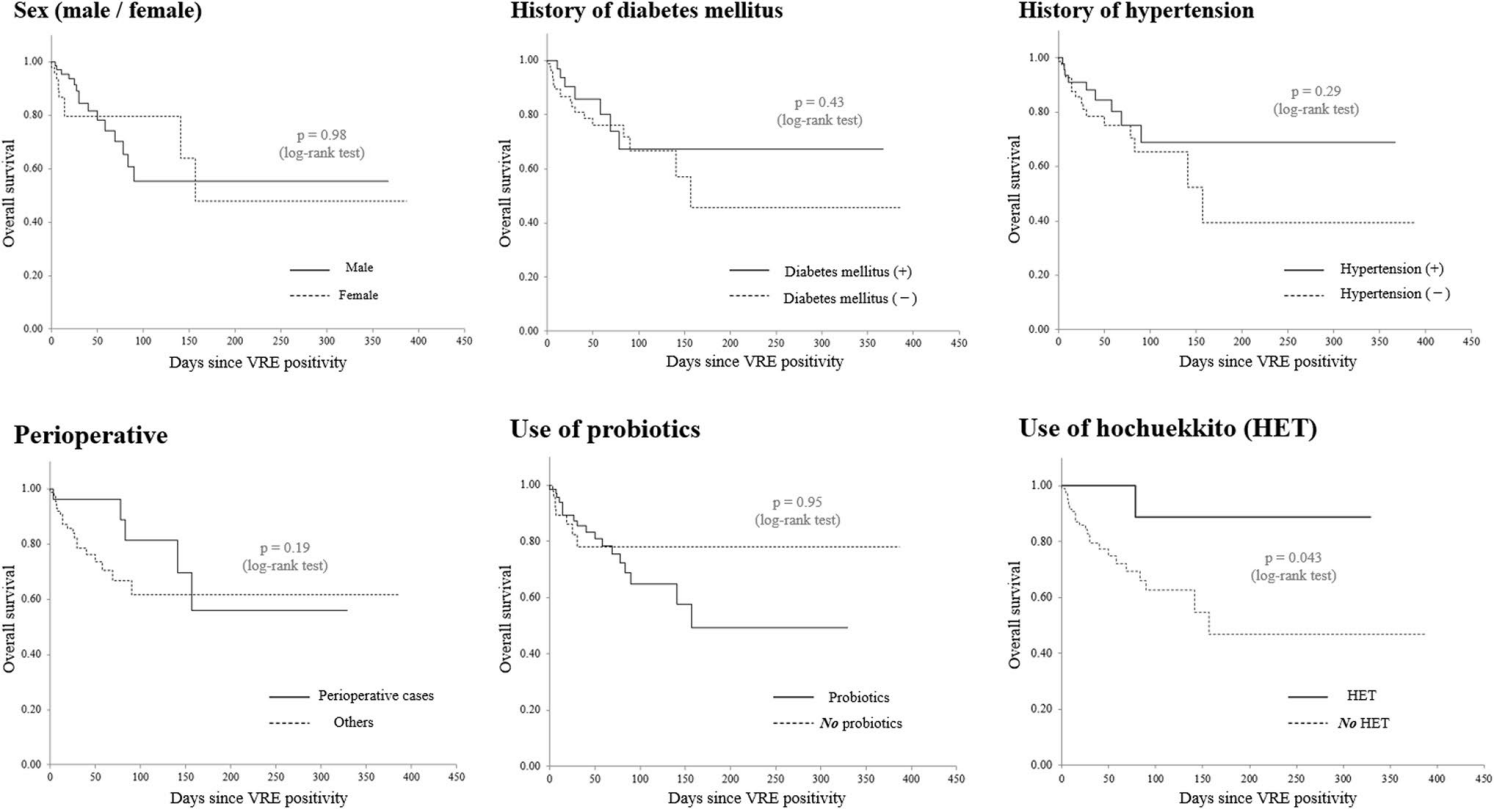
Overall survival analysis with each clinical and therapeutic variable. *p*-values were obtained by log-rank tests. The use of hochuekkito for the treatment of vancomycin-resistant enterococcus significantly contributed to a better overall survival. Meanwhile, the presence of diabetes mellitus or hypertension, perioperative status, probiotics, and sex did not significantly affect overall survival in this cohort.

Univariate survival analyses were then performed to evaluate the effect of each clinical and therapeutic variable on the overall survival rate. Kaplan–Meier curves, with *p*-values determined by log-rank tests for each demographic and clinical factor, are shown in Fig. 1. Sex, presence of diabetes mellitus or hypertension, peri-operational status, and use of probiotics did not significantly affect mortality. However, the administration of Kampo medicines significantly contributed to the survival rate of patients with VRE colonization (*p* = 0.031).

### Rate of achievement of VRE negative conversion

Since patients’ survival rates were improved by the subsequent achievement of VRE negative conversion, we investigated the factors affecting the rate of VRE negative conversion with a univariate analysis. In particular, the following factors were assessed: history of diabetes mellitus, hypertension, and dyslipidemia; perioperative status; preceding use of antibiotics before confirming VRE in the stool; and therapeutic administration of probiotics or Kampo medicines. The results of the univariate analysis are shown in Table 2. The administration of probiotics (*p* = 0.0065, n = 69) and Kampo medicines (*p* = 0.0002, n = 18), including HET (*p* = 0.0014, n = 16), positively contributed to VRE negative conversion. Moreover, a higher serum albumin level at hospitalization contributed to a higher achievement rate of VRE negative conversion; the other evaluated factors did not contribute to this.

**Table 2:** Comparative analysis of clinical and therapeutic parameters according to the subsequent achievement of VRE negative conversion.

	Subsequent achievement of VRE negative conversion		*p*-value
	Yes (n = 39)	No (n = 83)	
Male:Female (n)	23:16	50:33	0.8941
Age (years)*	71.9 ± 12.4 years	72.1 ± 14.0 years	0.9552
Body mass index at hospitalization*	23.22 ± 4.85	21.93 ± 4.09	0.1491
Serum albumin level at hospitalization*	3.37 ± 0.85 g/dL	3.02 ± 0.79 g/dL	0.0310
**Past medical history**			
Diabetes mellitus, n (%)	10 (25.6%)	28 (33.7%)	0.3680
Hypertension, n (%)	17 (43.6%)	33 (39.8%)	0.6883
Dyslipidemia, n (%)	7 (17.9%)	6 (7.2%)	0.1125
**Details of treatment after admission before VRE detection**			
Perioperative case (n; surgery after admission [%])	10 (25.6%)	18 (21.7%)	0.6281
Use of antibiotics (total; n [%])	34 (87.2%)	76 (91.6%)	0.5186
Use of antibiotics (VCM; n [%])	6 (15.4%)	20 (24.1%)	0.3466
Use of a nasogastric feeding tube, n (%)	21 (53.8%)	33 (39.8%)	0.1440
**Details of the treatment for VRE**			
Probiotics, n (%)	29 (74.4%)	40 (48.2%)	0.0065
Total Kampo medicines, n (%)	13 (33.3%)	5 (6.0%)	0.0002
Hochuekkito, n (%)	11 (28.2%)	5 (6.0%)	0.0014

Kampo medicines (total) means hochuekkito (n = 16), juzentaihoto (n = 1), and ninjin'yoeito (n = 1).

*VCM* vancomycin, *VRE* vancomycin-resistant enterococcus.

*Mean and standard deviation.

### Rate of achievement of VRE negative conversion

Next, we conducted multivariate binary logistic regression analysis to examine the rate of achievement of VRE negative conversion. We included the following factors as explanatory variables: history of diabetes mellitus, body mass index at hospitalization, serum albumin level at hospitalization, use of a nasogastric feeding tube, and treatment with HET. The results of the multivariate analysis are shown in Table 3. The use of HET (odds ratio: 8.477, 95% confidence interval [CI]: 2.054–34.980, *p* = 0.0031) positively contributed to the achievement of VRE negative conversion. Body mass index at hospitalization (1.109, 95%CI: 1.001–1.229, *p* = 0.0473) also contributed to the subsequent achievement of VRE negative conversion, whereas the remaining variables did not.

**Table 3:** Binary logistic regression analysis for the subsequent achievement of VRE negative conversion.

	B	SEB	Wald	OR (95% CI)	*p*-value
(Constant)	− 4.868	1.608	9.170	0.008 (0.000–0.180)	0.0025
History of diabetes mellitus	− 0.955	0.538	3.150	0.385 (0.134–1.105)	0.0759
BMI at hospitalization	+ 0.104	0.052	3.934	1.109 (1.001–1.229)	0.0473
Serum albumin level at hospitalization	+ 0.466	0.292	2.560	1.594 (0.900–2.823)	0.1096
Use of a nasogastric feeding tube	+ 0.489	0.470	1.084	1.631 (0.649–4.097)	0.2977
Treatment with hochuekkito	+ 2.137	0.723	8.734	8.477 (2.054–34.980)	0.0031

All independent variables were treated as ordinal variables. OR = exp(B), Wald χ^2^ statistics (Wald) = ${\left(B/SEB\right)}^{2}$. Wald is a marker of the significance of each coefficient in the applied predictive model.

*B* unstandardized partial regression coefficient, *BMI* body mass index, *CI* confidence interval, *OR* odds ratio, *SEB* standard error of the coefficient, *VRE* vancomycin-resistant enterococcus.

### Time until VRE negative conversion

**Figure 2:**
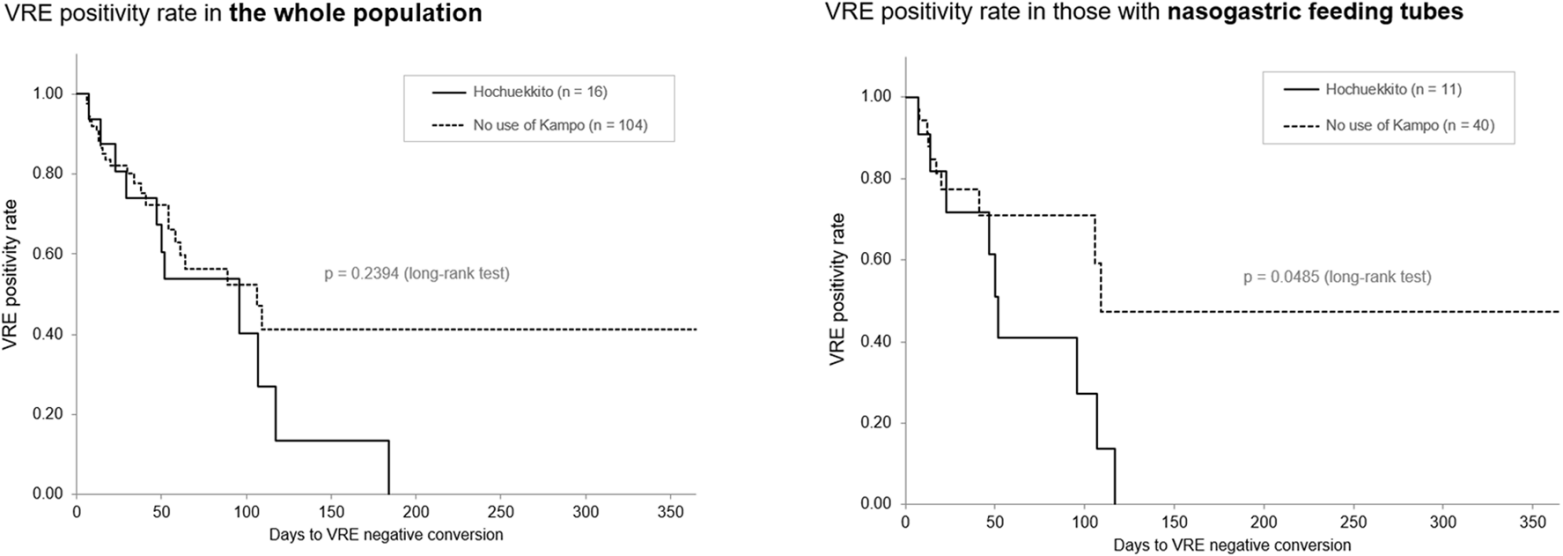
Kaplan–Meier curves until VRE negative conversion by hochuekkito. Time until the achievement of VRE negative conversion was compared between patients treated with hochuekkito and others without use of any Kampo medicine in the whole population and in those with nasogastric feeding tubes. *VRE* vancomycin-resistant enterococcus.

Lastly, based on the results that suggested the positive effect of HET in patients with VRE, we further investigated the time differences until VRE negative conversion between those treated by HET and those with no use of Kampo medicine. The Kaplan–Meier curves for the time until the achievement of VRE negative conversion in the whole population and in those nourished via nasogastric feeding tubes are shown in Fig. 2. In the whole population, the period until VRE negative conversion was not significantly different between the two therapeutic groups (HET: n = 16 vs. no use of Kampo: n = 104; *p* = 0.2394, log-rank test), but it showed differences in those of similar nutrition status with nasogastric feeding tubes (n = 11 vs. n = 40; *p* = 0.0485).

## Discussion

In the present study, we demonstrated that the mortality of VRE-positive patients was affected by VCM use and subsequent VRE negative conversion. Use of VCM indicated the immune-compromised status of patients suffering from MRSA infection, and the failure of VRE negative conversion indicates the severity of the patient’s condition. The administration of Kampo medicines contributed to the achievement of VRE negative conversion in uni- and multivariate analyses. Moreover, Kampo treatment significantly contributed to the speed of VRE negative conversion. Administration of probiotics also contributed to the achievement of VRE negative conversion but did not affect its speed.

The risk of VRE infection depends on a patient’s nutritional intake, immune status, the condition of the intestinal flora, and the patient’s history of antibiotic use. The use of probiotics for eliminating VRE infections is debated^4–6^, but it has been suggested that probiotics promote a healthy intestinal environment^14^. The present study indicates that probiotics may promote VRE negative conversion, but the combined treatment of probiotics and Kampo medicines showed a stronger and faster effect for the achievement of negative conversion. A higher serum albumin level and body mass index, which reflect the nutritional status, positively contributed to the subsequent VRE negative conversion in the present study. Malnutrition is also an independent factor associated with nosocomial infections^15^. HET is a Kampo medicine originally used to promote digestion and absorption and improve the nutritional status by reducing exhaustion caused by inflammation^16^. Combined administration of Kampo medicines and probiotics may promote the health of the intestinal environment, as well as digestion and absorption, resulting in an improved nutritional condition. Further study will be needed to investigate the nutritional status related with the administration of Kampo medicine and the contribution to VRE decolonization.

Few studies assessed the effects of Kampo medicines for multidrug-resistant bacteria. Previous studies have indicated that HET has anti-inflammatory effects on the intestinal mucosa, promotes recovery of the immune system, and increases appetite^16^. JTT and NYT also have similar effects^17^. HET, JTT, and NYT contain common crude drugs: The Japanese Pharmacopoeia (JP), Astragalus Root, JP Ginseng, JP Japanese Angelica Root, JP Atractylodes Lancea Rhizome, and JP Glycyrrhiza (Table 4). Combinations of these drugs may be key for cytokine regulation, activation of innate immunity, and healing in compromised patients^16,17^. Therefore, we can conclude that Kampo medicines can combat VRE colonization in immunocompromised patients through multiple mechanisms.

**Table 4:** Composition of the three Kampo formulae used in this study.

HET	(g)	JTT	(g)	NYT	(g)
**JP Astragalus Root**	4	**JP Astragalus Root**	3	**JP Astragalus Root**	1.5
**JP Ginseng**	4	**JP Ginseng**	3	**JP Ginseng**	3
**JP Japanese Angelica Root**	3	**JP Japanese Angelica Root**	3	**JP Japanese Angelica Root**	4
**JP Atractylodes Lancea Rhizome**	4	**JP Atractylodes Lancea Rhizome**	3	**JP Atractylodes Rhizome**	4
**JP Glycyrrhiza**	1.5	**JP Glycyrrhiza**	1.5	**JP Glycyrrhiza**	1
JP Bupleurum Root	2	JP Poria Sclerotium	3	JP Poria Sclerotium	4
JP Jujube	2	JP Rehmannia Root	3	JP Rehmannia Root	4
JP Citrus Unshiu Peel	2	JP Cinnamon Bark	3	JP Cinnamon Bark	2.5
JP Cimicifuga Rhizome	1	JP Peony Root	3	JP Peony Root	2
JP Ginger	0.5	JP Cnidium Rhizome	3	JP Polygala Root	2
				JP Citrus Unshiu Peel	2
				JP Schisandra Fruit	1

The formulae contained the following inactive ingredients: JP Magnesium Stearate and JP Lactose Hydrate.

The crude drugs in bold are common in all three formulae.

HET is indicated for patients with a delicate constitution, reduced digestive function, and severe fatigability of the limbs: summer emaciation, reinforcement of physical strength after illness, tuberculosis, anorexia, gastroptosis, cold, hemorrhoid, anal prolapse, uterine prolapse, impotence, hemiplegia, and hyperhidrosis. JTT and NYT are indicated for the relief of weakness after recovery from disease, fatigue, malaise, anorexia, perspiration during sleep, cold limbs, and anemia.

*HET* hochuekkito, *JTT* juzentaihoto, *NYT* ninjin'yoeito, *JP* The Japanese Pharmacopoeia.

In infectious diseases, the efficacy of prebiotics and probiotics has been reported. Guadalupe et al. reported an association between the administration of probiotics combined with prebiotics and the prevention or modulation of severe rotavirus gastroenteritis^18^. Rätsep et al. reported that symbiotic use during antimicrobial therapy can prevent *C. difficile* infection, as well as its potential to reduce recurrences^19^. Kampo medicines have been proven to influence the microbiome; thus, Kampo medicines have prebiotic effects^20,21^. The bioactive constituents of HET, including astragaloside IV, calycosin, glycyrrhizic acid, enoxolone, saikosaponin D, ferulic acid, and hesperidin, are distributed in the gastrointestinal tract^22^ and can influence the gastrointestinal tract condition together with the microbiome. HET includes triterpenoid saponins, which undergo deglycosylation in a metabolic pathway related to the microbiome^23^. In our study, the effect of Kampo medicines was promoted by probiotics, as supported by the metabolism of saponin to aglycon, which is mediated by probiotics^24,25^. These reports may explain the additional effects of probiotics on Kampo medicines in the gut environment and immune system.

In the Kampo theory, HET, JTT, and NYT are considered to belong to the Ginseng Root and Astragalus Root drug group, which improve body energy. They are traditionally used to relieve weakness after recovery from a disease, fatigue and malaise, and anorexia, and listed in some clinical practice guidelines in Japan^26–31^. HET is indicated for patients with a delicate constitution, reduced digestive function, and severe fatigability of the limbs. JTT and NYT are indicated for the relief of weakness after recovery from disease, fatigue, anorexia, perspiration during sleep, cold limbs, and anemia, and NYT is indicated for patients with symptoms such as cough or insomnia. Since these three Kampo medicines contain Glycyrrhiza, they can cause pseudoaldosteronism. Patients should be carefully monitored to prevent the reduction of serum potassium levels, increased blood pressure, and edema. Other side effects, such as hepatic dysfunction and interstitial pneumonia, were also reported. If these side effects are observed, ceasing the administration of Kampo medicines and administering general medications for adverse events could improve symptoms. In the present study, one patient treated with Kampo medicines showed hypokalemia but soon improved with the administration of spironolactone. Considering these side effects, we think it is safe to introduce Kampo medicines.

In Japan, linezolid and Quinopristin-Dalfopristin are currently approved by the Japanese government for the treatment of VRE infections. Daptomycin is also recommended for VRE infections^3^, but decolonization is still important to reduce VRE spread, decrease the risk of infection, and avoid acquiring tolerance to such anti-VRE drugs.

Nosocomial VRE infections may cause death among immunocompromised patients. The present study indicates that Kampo medicines may contribute to reducing VRE carriers and severe infections, thus reducing the risk of VRE infections in vulnerable patients.

There are some limitations to the present study. As it had a retrospective observational design, participant selection was not precisely controlled. Not all VRE genotypes were examined, and other genotypes could have been present. We did not collect data on the specific antibiotics administered. Furthermore, we did not survey the use of proton pump inhibitors. Further data are needed to investigate the contribution of these factors to VRE decolonization in Japanese patients.

In conclusion, the therapeutic use of Kampo medicines in addition to probiotics positively contributed to the higher rate and speed of VRE negative conversion. The Kampo medicines in this study may aid in controlling antibiotic-resistant bacterial nosocomial infections.

## Methods

### Patients

This study assessed consecutive patients with positive VRE in culture examination from a tertiary-care hospital located in the Aomori prefecture of Japan from August 2018 to July 2019. The hospital has an advanced emergency medical center that receives 24,000 emergency patients every year and has 30 beds in the emergency unit. Once the first VRE-positive patient was identified in August 2018, the infection spread from the emergency unit and intensive care unit to other hospital wards. During this period, the hospital examined the stool cultures of all inpatients every week, and the data of all VRE-positive patients were listed by the clinical laboratory department. A total of 122 inpatients whose culture samples were confirmed to be VRE-positive were enrolled in this study. With respect to the list of VRE-positive patients, we retrospectively collected the following data from hospital medical records.

### VRE detection

Fecal samples were obtained by collecting swabs (E-MS60, EIKEN Co., Ltd, Tochigi, Japan). Then, samples were cultured on VRE-selective agar (BD BBLTM, Cat. No.251833, Becton, Dickinson, and Company, NJ, USA). If colonies grew, they were further cultured and VCM resistance examined by BD Sensi-Disc, Va30 (Cat. No. 291019, Becton, Dickinson, and Company, NJ, USA). The minimal inhibitory concentration (MIC) was determined using PC1J panel with MicroScan WalkAway96Plus (Beckman Coulter, Inc., CA, USA). VCM resistance was considered at an MIC of > 16 μg/mL. When VRE were detected, their genotype was examined by the Aomori prefecture public health and environmental center using the pulsed-field gel electrophoresis method. We defined VRE negative conversion to be achieved if the colony could not be detected three times consecutively on VRE-selective agar. The date of VRE negative conversion was defined as the date of sample collection for the third culture test among the three consecutive times of negative colony growth in VRE-selective agar.

### Studied variables

For the 122 VRE-positive patients enrolled, the following demographic and clinical data were collected: sex, age, body mass index at hospitalization, serum albumin level at hospitalization, history of diabetes mellitus, history of hypertension, surgical operation after hospitalization, and preceding use of antibiotics for treating bacterial infections before confirming VRE positivity in the stool. In addition to these pre-onset factors, the following therapeutic factors were also evaluated: use of oral probiotics (n = 69) and Kampo medicines (n = 18). The outcomes evaluated were the eventual achievement of negative conversion of VRE (binary variable) and the time between confirming VRE positivity in the stool and achievement of VRE negative conversion (continuous variable).

Since February 2019, the hospital recommended that VRE-positive patients be treated with a probiotic formula to improve enteric microbial balance. Prescription of probiotic formulae, selection of formulation, combined use or not, dosage, and duration of administration were based on the attending doctors’ decision. The species of probiotic formulae available at the hospital were the following: BIO-THREE (*Enterococcus faecium*, *Clostridium butyricum,* and *Bacillus subtillis*), LAC-B GRANULAR POWDER N (*Bifidobacterium longum* and *Bifidobacterium infantis*), MIYA-BM FINE GRALULES (*Clostridium butyricum*), and BIOFERMIN POWDER (*Streptococcus Faecalis* and *Bacillus subtilis*).

As for the 18 patients treated with Kampo medicines, 16 were treated with 7.5 g of HET in three doses per day, 1 with 7.5 g of JTT in three doses per day, and 1 with 9.0 g of NYT in three doses per day. A Kampo specialist and doctors consulting him prescribed these Kampo medicines to VRE-positive patients. The selection of the Kampo formula was as follows: for patients with some diseases and a vulnerable status, the first choice was HET. If the patient was anemic; red blood cells < 3.7 × 10^6^ /μL, hemoglobin < 11.5 g/dL, and hematocrit < 35%, JTH was selected. If patients suffered from anemia and respiratory diseases, NYT was prescribed.

Table 4 shows the composition of the three Kampo formulae used in this study. Further information regarding these Kampo formulae can be found on the website of the Standards of Reporting Kampo Products^32^. All Kampo formulae used during the study were manufactured by Tsumura and Co. (Tokyo, Japan).

### Statistical analysis

Distributions of normally distributed numeric variables are described as means and standard deviations, and those of non-normally distributed numeric variables as medians and interquartile ranges (25–75 percentiles). Comparisons between two variables were performed by using either Student’s t-test or Mann–Whitney’s U-test according to the variables’ distribution pattern. Survival analysis was assessed by Kaplan–Meier curves and the log-rank test. Multivariate analysis for the subsequent achievement of VRE negative conversion was assessed by binary logistic regression analysis with explanatory variables of particular clinical interest and additional variables that had a significant impact (*p* < 0.10) on the achievement of VRE negative conversion in univariate analyses. In multivariate analysis, explanatory variables with a moderate-to-strong correlation were not simultaneously employed to avoid multicollinearity. In each analysis, *p*-values < 0.05 were considered statistically significant. When multiple comparisons were simultaneously performed, *p*-values < 0.01 were considered statistically significant by applying the Bonferroni correction method. Statistical analyses were performed using SPSS Statistics Base 22 software (IBM Corp., Armonk, NY, USA) or MATLAB R2015a (MathWorks, Natick, MA, USA).

### Ethics declarations

This study was approved by the Institutional Review Board of Hachinohe City Hospital (Institutional Review Board approval number: 1933) and performed in accordance with the ethical standards of the 1964 Declaration of Helsinki. The institutional review board waived the requirement for informed consent due to the retrospective study design.

## Data Availability

The datasets analyzed during the current study are available from the corresponding author on reasonable request.
